# Mandatory TB notification in Mysore city, India: Have we heard the private practitioner’s plea?

**DOI:** 10.1186/s12913-016-1943-z

**Published:** 2017-01-03

**Authors:** Sarabjit Singh Chadha, Sharath Burugina Nagaraja, Archana Trivedi, Sachi Satapathy, Devendrappa N M, Karuna Devi Sagili

**Affiliations:** 1 International Union against Tuberculosis and Lung Disease, South-East Asia Regional Office, Qutub institutional area, Hauz Khas, New Delhi 110016 India; 2 Department of Community Medicine, ESIC Medical College and PGIMSR, Rajajinagar, Bangalore, Karnataka 560010 India

**Keywords:** TB Notification, Private sector, NIKSHAY, India, Tuberculosis

## Abstract

**Background:**

The Government of India, made TB notification by private healthcare providers mandatory from May 2012 onwards. The National TB Programme developed a case based web based online reporting mechanism called NIKSHAY. However, the notification by private providers has been very low. We conducted the present study to determine the awareness, practice and anticipated enablers related to TB notification among private practitioners in Mysore city during 2014.

**Methods:**

A cross-sectional study was conducted among private practitioners of Mysore city in south India. The private practitioners in the city were identified and 258 representative practitioners using probability proportional to size were interviewed using semi-structured questionnaire.

**Results:**

Among the 258 study participants, only 155 (60%) respondents agreed to a detailed interview. Among those interviewed, 141 (91%) were aware that TB is a notifiable disease; however 127 (82%) of them were not aware of process of notification and NIKSHAY. Only one in six practitioners was registered in NIKSHAY, while one in three practitioners are notifying without registration. The practitioners expected certain enablers from the programme like free drugs, training to notify in NIKSHAY and timely feedback. 74 (47%) opined that notification should be backed by legal punitive measures.

**Conclusion:**

The programme should develop innovative strategies that provide enablers, address concerns of practitioners while having simple mechanisms for TB notification. The programme should strengthen its inherent capacity to monitor TB notification.

**Electronic supplementary material:**

The online version of this article (doi:10.1186/s12913-016-1943-z) contains supplementary material, which is available to authorized users.

## Background

Globally, tuberculosis (TB) remains a major public health problem and ranks second among the leading causes of death due to infectious disease. In 2015, there was an estimated 9.6 million new TB cases world-wide of which an estimated 2.2 million occurred in India representing 23% of the global TB burden [1].

Across the globe, nearly three million TB cases are estimated to be missed by national notification systems, of which nearly one million are in India [1]. Until recently, only those TB cases that were managed by the public health system were notified in India; while the private health sector had no obligation to notify a TB case. A community based survey conducted in 2011 reported that 46% of the TB patients in India were treated outside the public health system (private sector) and hence are not part of the national TB notification system [2]. In May 2012, Government of India issued a gazette notification which makes it mandatory for private practitioners to notify any case of TB that they diagnose or treat. The mechanisms provided for notification include both paper and case based web based online reporting system called NIKSHAY. If a private practitioner wishes to notify a case of TB, the practitioner has to either send the list of patients in the paper reporting format prescribed by the programme to the district TB officer or has to enter the data directly into NIKSHAY. Initially, for notification directly through NIKSHAY the practitioners have to do a one-time self-registration before they choose to notify TB patients. However, there remains a lacuna to monitor and ensure notification by private practitioners while there are no regulatory measures if the practitioner do not notify.

Despite the government gazette, TB notification by private practitioners is sub-optimal. According to the annual TB report of India 2014, only about 30,000 cases (~3% of total notified cases)were notified from the private sector [3]. The non-notification of TB cases not only leads to under-estimation of the patients with TB morbidity and mortality but also impairs the country’s strategic planning for control of TB. Notification also helps private practitioner to monitor their patients’ compliance to treatment under the programme. It is deemed necessary for policy makers and programme managers to have an insight into the private care provider’s awareness, perception, practice, mechanisms and enabling factors for TB notification which is essential to strategize and strengthen TB notification in the country. Hence, we conducted a study to determine the practice, mechanism and enabling factors for TB notification among private practitioners in Mysore city, Karnataka, India.

## Methods

The study was conducted in Mysore city of Mysore district during March 2014 to August 2014. The district is located in the southern part of Karnataka state, South India with a population of nearly four million. The health care services in the district are delivered by private and public sector.

### Study design and study population

This was a cross sectional study and the study population included all the practitioners working in the private health sector of Mysore city. The private health sector included facilities like clinics, nursing homes, non-governmental (NGO) hospitals, corporate hospitals, private medical colleges and clinical laboratories.

### Sampling and sample size

All the qualified private practitioners in Mysore city were mapped by trained social workers and formed the sampling frame. All those practitioners who had a medical (allopathic) degree obtained from recognized health universities with or without specialization were considered as qualified private practitioners. The qualification of the practitioners was ascertained from the display boards outside their clinic or hospital. A total of 1249 qualified private practitioners were working in different types of private health facilities in the city (Table 1). A sample size of 258 was estimated with frequency of 20% notifying using NIKSHAY, 80% power, 5% of significance, design effect of 1 and considering 5% non-responders (unwilling to participate in the study). Probability proportional to size sampling technique was applied for each stratum (based on the type of health facilities) to obtain the desired sample size and systematic sampling was done from the line list to identify study participants.

**Table 1:** Distribution of practitioners at different type of private health facilities, Mysore, Karnataka, 2014 (*N* = 1249)

Type of Health Facilities	*N* (%)
a) Private Allopathic Clinics	389 (31)
b) Private Non-Allopathic (AYUSH)	104 (8)
c) Private Nursing Homes	204 (16)
d) Private Clinical Laboratories	46 (4)
e) Corporate Hospitals	147 (12)
f) Private Medical College	284 (23)
g) Private Dental Clinics	75 (6)

### Data collection, sources of data and study variables

The selected individual private practitioners were interviewed by trained social workers at their work place using semi-structured questionnaire after obtaining their consent (Additional file 1). A maximum of three visits to the practitioners were made to gather the information. The study variables that were collected included some socio-demographic variables like age, sex, qualification, years of experience and specific information in the past 3 months regarding TB notification like awareness, practice and anticipated enablers from the programme. The variable ‘enablers’ was introduced to gather information on incentives or enablers that could encourage practitioners to notify. An open ended question was designed for the variable enablers for whom more than one response was possible; for analysis the answers were grouped into major areas and measured in terms of proportions.

### Data entry and analysis

Data was entered into a structured format created on EpiData version 3.1 and analyzed using EpiData analysis version 2.2.178 (The EpiData Association, Odense, Denmark). The data was checked for duplicate records and cross verified before subjecting it to analysis. All variables were looked for proportions.

## Results

Of the 258 study participants, only 155 (60%) respondents agreed to a detailed interview while the remaining 103 (40%) did not participate. They cited the following reasons: all the TB cases are referred to government health facilities (88, 85%) and/or they do not diagnose or treat TB (64, 62%) (Table 2). Awareness, practice and anticipated enablers of practitioners are shown in Table 3.

**Table 2:** Practitioners related reasons for not participating in the study, Mysore, Karnataka, 2014 (*N* = 103)

S.No	Reasons	*N* (%)
1	We generally do not diagnose or treat TB cases^a^	64(62)
2	We are specialists and do not deal with TB cases^a^	37(36)
3	We refer all TB cases to Government hospitals, we do not treat TB patients^a^	88(85)
4	Could not interact to practitioners as they were busy	2(2)
5	Practitioners not present at health facilities	3(3)
6	Our privacy will be intruded^a^	35(34)

^a^multiple responses

**Table 3:** Awareness, practice and enablers anticipated from practitioners on TB notification, Mysore, Karnataka, 2014 (*N* = 155)

2.1 Demography	*N* (%)
1 Sex	
a) Male	104 (67.1)
b) Female	51(32.9)
2 Age group	
a) <45 years	61(39.4)
b) ≥45 years	94(60.6)
3 Qualification	
a) MBBS	72(46.5)
b) Post MBBS Specialization	83(53.5)
4 How many TB patients do you diagnose?	
a) Nil	26(16.8)
b) 1 to 3	60(38.7)
c) >3	36(23.2)
d) Not recorded	33(21.3)
2.2 Awareness	
1 TB notifiable disease	
a) Yes	141(91.0)
b) No	14(9.0)
2 Source of information	
a) Advertisements	30(19.4)
b) RNTCP personnel’s	82(52.9)
c) Authorities	25(16.1)
d) Others	4(2.6)
e) Not recorded	14(9.0)
3 Is Notification compulsory?	
a) Yes	130(83.9)
b) No	25(16.1)
4 Heard of NIKSHAY?	
a) Yes	24(15.5)
b) No	127(81.9)
c) Not recorded	4(2.6%)
2.3 Practice	
1 Are you registered in NIKSHAY?	
a) Yes	24(15.5%)
b) No	127(81.9%)
c) Not recorded	4(2.6%)
2 Have you notified in NIKSHAY?	
a) Yes	7(4.5%)
b) No	138(89.0%)
c) Not recorded	10(6.5%)
3 Number notified in NIKSHAY?	
a) Nil	126(81.3%)
b) 1 to 3	5(3.2%)
c) >3	2(1.3%)
d) Not recorded	22(14.2)
4 Reasons for not registering/notifying?	
a) Suspect the motive	14(9.0)
b) Fear of losing patient	3(1.9)
c) Confidentiality	2(1.3)
d) Do not have patients	12(7.7)
e) Others	24(15.5)
f) No response	100(64.5)
2.4 Enablers	
1 Kindly mention the anticipated support?	
a) Training to register and notify	35(22.6)
b) Health Workers to collect data	35(22.6)
c) Provide free drugs	43(27.7)
d) feedback/acknowledgement on notification	34(21.9)
e) Others	2(1.3)
f) Not recorded	6(3.9)
2 Seeking written declaration by the private practitioner?	
a) Totally unnecessary	17(11.0)
b) Unnecessary	19(12.3)
c) No comment	42(27.1)
d) Necessary	77(49.7)
3 Action to be taken against practitioners?	
a) Totally unnecessary	13(8.0)
b) Unnecessary	27(17.4)
c) No comment	41(26.5)
d) Necessary	72(46.5)
e) Not recorded	2(1.3)
4 Should be backed by legal punitive measures?	
a) Totally unnecessary	12(7.7)
b) Unnecessary	18(11.6)
c) No comment	47(30.3)
d) Necessary	74(47.7)
e) Not recorded	4(2.6)

### Profile of practitioners

Majority of the respondents (104 out of 155, 67%) were males and aged above 45 years (94 out of 155, 60%). 83 (53%) respondents had done specialization after completing the basic medicine course. 96 (62%) respondents said that they manage TB (diagnose and/or treat) and 60 (38%) of them had diagnosed at least one to three TB cases in the last 3 months.

### Awareness

Majority of the respondents (141 out of 155, 91%) were aware that TB is now a notifiable disease and the main source of information was through the National TB Programme staff (82 out of 155, 53%) followed by advertisements (30 out of 155, 19%), 130 (83%) respondents knew that notification was compulsory and 127 (82%) were unaware of NIKSHAY.

### Practice

Only 24 (15.5%) of the respondents were registered in NIKSHAY and only 7 out 24 (29%) had notified in NIKSHAY. When all the 155 practitioners were posed with a question of reasons for not registering or notifying TB cases, only 55 opted to answer. The major reasons were: 25% (14 out of 55) were suspicious of the motive behind notification; 22% (12 out of 55) did not treat TB patients during the period and the remaining were worried about losing their patients and breaching their patients’ confidentiality.

### Enablers

The anticipated enablers for TB notification were (a) providing anti-tubercular drugs free of cost to the patients (43 out of 155,28%) (b) Training to the practitioners to notify in NIKSHAY (35 out of 155, 23%) (c) providing feedback after notification (35 out of 155, 22%) and (d) regular support from health workers to collect data. The most common response (74 out of 155, 47%) was initiating legal punitive measures against those who fail to notify.

## Discussion

It is one of the fewer studies conducted to determine the awareness, practice and anticipated enablers for TB notification among private practitioners in India. The study reveals very important findings which have programmatic implications as discussed below.

Firstly, nearly 40% of the practitioners did not agree to be part of the detailed interview citing that they did not manage TB cases. This could be attributable to the limited awareness about the objective behind notification leading to misconceptions and suspicion in the practitioner’s minds which was mentioned during the interviews. One of the fears related to notification could be the potential use of this information to audit the diagnostic and treatment practices of practitioners which have been found to be sub-optimal in various studies [4]. The practitioners also mentioned breaching patients’ confidentiality as a reason for not notifying which probably stems from the widely prevalent stigma related to TB [5, 6].

These misconceptions and apprehensions have to be addressed by the programme for which, the possible measures could be (a) continuous interaction with the practitioners on a one to one basis by the district TB officer or the medical officers which clarifies their queries. This is of high importance in terms of gaining the trust and confidence from the practitioners (b) partnering with non-governmental organizations, professional bodies and other partners for educating the practitioners through meetings and sensitization programmes.

Secondly, among the practitioners interviewed, majority of them were well aware about TB notification being mandatory and the main source of information was from the National TB Programme staff. During May 2012, the programme issued a notification on notifying TB. Initial efforts of dissemination were made from Ministry of Health and Family Welfare through newspaper advertisements and the district programme staff. The staffs were directed to make concrete efforts to communicate the circular of TB notification to all health care institutions and private practitioners in the district. An informal review of the Government notification and the published advertisement shows that the objective behind TB notification was not clarified adequately. The programme has to revise its communication strategy to have an impact on practitioners. The programme should also expand its awareness campaign to educate the general public to dispel stigma and educate TB patients regarding mandatory notification and assure them that their confidentiality will be maintained. The awareness generation should not be restricted to a one time activity. While there should be pulsatile events, dynamic strategies need to be devised to evoke public and practitioners’ interest. Advocacy model at districts involving medical associations and private practitioners engaged in TB notification to motivate their peer groups should be considered. The policy makers should envisage on involving other sectors by developing schemes for their involvement. This includes non-governmental organizations, panchayat raj institutions (A governing body formed by elect members at village level), educational institutions and corporate sectors. India’s experience drawn from successful pulse polio programme suggests that it is worth considering a ‘TB notification drive’ in campaign mode.

Thirdly, the programme needs to take steps on some of the pertinent suggestions made by the practitioners to encourage them to notify. Foremost is the demand for training on how to notify which will help them understanding the notification process and use of NIKSHAY. Importantly, none of the practitioners opined that TB notification should be incentivized which reflects the zeal of the practitioners to participate in the programme for a social cause. Thus, the programme should strengthen its training capacity by delegating the training component to medical colleges. The programme has constituted RNTCP core committee at each of the medical colleges whose responsibility is to implement the programme at their institution. Also, the committee members are actively involved towards conduct of sensitization programmes for hospital staff, continued medical education for private practitioners, facilitators for government health TB training programmes and conduct of operational research apart from teaching medical students. The faculty of medical college has high potential to drive the notification campaign from the front. At the field level, the implementation of this newer initiative calls for increasing the human resource base of the programme; hence, a separate cadre of qualified paramedical staff in urban areas who would regularly visit the practitioners, support them in collecting data, facilitate the diagnosis and treatment of TB patients for at least the next 5 years poses to be the pragmatic solution. The programme needs to develop simple user friendly mechanism to report TB notification [7].

Providing free anti-TB drugs to practitioners for the patients will be a good approach promoting both notification and rational treatment while reducing out of pocket expenditure of the patient. Notification should not be a one way process and there should be a process of acknowledging and providing necessary feedback to the notifying practitioner, which does not currently happen.

Fourth, the practitioners had varied opinion regarding legal action in case the practitioner failed to notify. The failure to notify could be done by collecting information about the practitioner and the patient from the retail pharmacies where the patients purchase anti-TB drugs. This information could be cross checked with NIKSHAY if the patient had been notified and necessary action can be taken if it was not the case. In Indian settings, it is worthwhile having legal punitive measures for TB notification in lines of pre-natal diagnostic technique (regulation and prevention of misuse) act,1994 [7, 8]. The act was enacted and brought into operation from 1st January, 1996, in order to check female foeticide. The act prohibits determination and disclosure of the sex of the foetus. The person who contravenes the provisions of this act is punishable with imprisonment and fine.

The strengths of our study were (a) it was conducted in routine programmatic settings and all the practitioners were mapped and included in the sampling frame which depicts the ground reality of programme implementation (b) written informed consent were obtained prior to the interview of practitioners. The interviews were conducted by trained personnel at the practitioners’ work place and hence the content of information is more authenticated. We have adhered to STROBE guidelines for reporting of observational studies in writing this manuscript [9]. (c) The practitioners were not provided any incentives for the interview and we believe that the information provided by them is unbiased. The limitations of the study were (a) a significant proportion of the practitioners refused to participate in the study making it less representative (b) few of the study questions included had multiple options and internally inconsistent answers which could have impacted the interpretation and results. However, these limitations were not a hindrance to draw the results and conclusions.

## Conclusion

The programme should develop innovative strategies that provide enablers, address concerns of practitioners while having simple mechanisms for TB notification. Efforts are to be made to back notification by legal punitive measures and the programme should strengthen its inherent capacity to monitor TB notification.

## Additional file

Additional file 1: The semi-structured questionnaire. (DOCX 14 kb)

## Abbreviations

MDR-TB: Multi-drug resistant tuberculosis
NGO: Non-governmental organisation
RNTCP: Revised National Tuberculosis Control Programme
STROBE: Strengthening the reporting of observational studies in epidemiology
TB: Tuberculosis
