# Supplementary material for: Mandatory TB notification in Mysore city, India: Have we heard the private practitioner’s plea?
Source: BMC Health Serv Res. 2017 Jan 3;17:1. doi: 10.1186/s12913-016-1943-z (PMC5209831; doi:10.1186/s12913-016-1943-z)
Supplement: Additional file 1: — The semi-structured questionnaire. (DOCX 14 kb) [file 12913_2016_1943_MOESM1_ESM.docx]

**Questionnaire**

**Title: Mandatory TB notification in Mysore city, India: Have we heard the private practitioner’s plea?**

| M | Y | S |  |  |  |  |  |
| --- | --- | --- | --- | --- | --- | --- | --- |

Unique number :

Name of the interviewer:

Date of interview:

Time of interview: Morning / afternoon / Evening / Night

Classification of practitioner: very busy / busy / average / relatively free ________________________________________________________________

**Physician's details**

Age : …………Years

Sex : Male / Female

Qualification : MBBS / MD or MS / Diploma / Others

Specialty : …………………………………………………

Experience : ………………Years

Area : Urban / Rural

1. Do you diagnose or treat TB? (If no, go to Q5) Yes / No

2. How many TB patients do you diagnose/treat in a month? _____

3. How many of the TB patients that you diagnose start the treatment with you? _____

4. How many TB patients you initiated on treatment complete the full treatment with you? _____

5. Have you heard that TB has been made a notifiable disease? Yes / No

6. If yes, what is the source of this information: paper ad / RNTCP personnels / Communication from authorities / specify _________________________________________________________________

7. Do you think that Notification of TB to government is compulsory? Yes / No

8. Have you heard of Nikshay? If no, go to question number 12. Yes / No

9. Are you registered on Nikshay? Yes / No

10. If yes, have you notified any TB patients on Nikshay? Yes / No

11. If yes, average numbers of TB patients notified in Nikshay? ______

12. Name three challenges you face in notification through Nikshay?

12.1.

12.2.

12.3.

13. Though registered, not notifying the TB patients? Reasons thereof;

a. The Govt only want to collect the information and will do nothing about this

b. Suspect the motive of the Government

c. Fear of losing patients

d .Patient confidentiality revealed

e. Do not have patients regularly

f. Others- please specify ________________________________________

14. How do you think the notification information should be/can be appropriately used for TB control?

14.1.

14.2.

14.3.

15. We would like to know your opinion regarding the following statements based on your experience

and the setting in which you work .

**Scoring: 1. Strongly Disagree; 2. Disagree; 3.No Opinion; 4.Agree; 5.Strongly Agree**

15.1. Notification gives an opportunity to Government to support private sector _____

15.2. Notification of TB helps patient to get right diagnosis __ ___

15.3. Notification of TB helps patient to get right treatment _____

15.4. Notification of TB of private sector patients to Government will help on follow up of patient _____

15.5. Notification of TB to Government will help in tracing contacts of TB patients. _____

15.6. Notification of TB to Government will help in giving chemoprophylaxis to contacts. __

15.7. Notification of TB to Government will help in facilitating social support for the patient in the community _____

16. Kindly mention the type of support, if expecting support (circle the appropriate)

a. Training

b. Health workers to come and collect details

c. Provision of free TB drugs from Government

d. Feedback to private regarding TB patient notified from institution

e. Others specify ………………………………………………………..

17. What is your opinion about giving a declaration by private doctor that they are not

Diagnosing/treating TB cases? (circle the appropriate)

Totally Unnecessary / Unnecessary / No comment / Necessary

18. What is your opinion regarding the action to be taken against doctors who treat TB and do not

notify the same?

Totally Unnecessary / Unnecessary / No comment / Necessary

19. Do you think TB Notification should be backed by legal punitive measures?

Totally Unnecessary / Unnecessary / No comment / Necessary
